# The Lived Experience of Mechanical Restraint: A Multicenter Phenomenological Study in Psychiatric Inpatients

**DOI:** 10.1192/j.eurpsy.2026.11294

**Published:** 2026-07-31

**Authors:** C. Spallarossa, C. M. Esposito, F. Altamore, P. Politi, C. Nichini

**Affiliations:** 1 Department of Brain and Behavioral Sciences, Università di Pavia, Pavia; 2 Ospedale Maggiore Policlinico, Fondazione IRCCS Ca’ Granda , Milano; 3 Azienda Usl Toscana centro, Firenze, Italy

## Abstract

**Introduction:**

Despite the progressive deinstitutionalization of mental health care, mechanical restraint remains an ethically controversial practice in psychiatry. Often justified as an *extrema ratio* to ensure safety, it conceals profound existential implications. A phenomenological perspective explores restraint not as a technical act but as a lived event. This study seeks to reveal the meanings attributed to restraint by patients, contributing to an ethically grounded reflection on coercion and care.

**Objectives:**

The study aimed to: (1) describe the subjective experience of restraint as narrated by psychiatric inpatients; (2) promote a phenomenological and ethically informed understanding of coercion, emphasizing how relational quality can mitigate or transform its traumatic impact.

**Methods:**

A multicentre qualitative study was conducted in seven psychiatric wards in Italy. Inpatients (N = 99; mean age = 37 ± 15 y; 37 women) who had undergone at least one episode of restraint completed an anonymous open-ended questionnaire within 72 hours. The prompt *“How would you describe the episode of restraint you experienced during this admission?”* elicited first-person narratives. Data were analysed using **Consensual Qualitative Research (CQR)**, integrating phenomenological description with consensus-based coding among multiple researchers and validation by an external auditor (Hill et al., *J Couns Psychol*, 2005). Ethical approval and informed consent were obtained.

**Results:**

Five dimensions structured the experience: **Time** was often suspended or fragmented (“*minutes felt eternal*”), revealing a rupture in lived temporality; **Space** shifted from a familiar world to a confining geometry of control, evoking claustrophobic imagery (“*like being inside a coffin but alive*”); **Body** emerged as both object and witness of suffering (“*my wrists are the stigmata of this admission*”), oscillating between pain and resistance; **Others** were perceived as punitive or distant, though rare gestures of care restored trust (“*she stayed with me and I felt safe again*”); **Selfhood** oscillated between loss of agency, rage, humiliation, and, retrospectively, partial reconciliation. Across domains, restraint appeared as a **liminal event** suspending ordinary being-in-the-world, exposing both trauma and residual possibilities of meaning.

**Conclusions:**

The impact of restraint depends less on the act itself than on the ethical and relational context surrounding it. When embedded in communication and empathy, coercive interventions may acquire reparative significance; when devoid of recognition, they risk becoming dehumanizing. Viewing restraint through lived experience enables psychiatry to face its “blind spot” - the coexistence of violence and care - and to transform coercion into a field of reflection rather than denial. Ultimately, each act of restraint tests the institution’s capacity to employ **listening as both an ethical and epistemic act**.

**Disclosure of Interest:**

None Declared

